# Calcium Imaging and the Curse of Negativity

**DOI:** 10.3389/fncir.2020.607391

**Published:** 2021-01-06

**Authors:** Gilles Vanwalleghem, Lena Constantin, Ethan K. Scott

**Affiliations:** Neural Circuits and Behavior Laboratory, Queensland Brain Institute, The University of Queensland, St Lucia, QLD, Australia

**Keywords:** calcium imaging, zebrafish, GCaMP, baseline fluorescence, data analysis, cerebellar circuitry, segmentation, spike inference

## Abstract

The imaging of neuronal activity using calcium indicators has become a staple of modern neuroscience. However, without ground truths, there is a real risk of missing a significant portion of the real responses. Here, we show that a common assumption, the non-negativity of the neuronal responses as detected by calcium indicators, biases all levels of the frequently used analytical methods for these data. From the extraction of meaningful fluorescence changes to spike inference and the analysis of inferred spikes, each step risks missing real responses because of the assumption of non-negativity. We first show that negative deviations from baseline can exist in calcium imaging of neuronal activity. Then, we use simulated data to test three popular algorithms for image analysis, CaImAn, suite2p, and CellSort, finding that suite2p may be the best suited to large datasets. We also tested the spike inference algorithms included in CaImAn, suite2p, and Cellsort, as well as the dedicated inference algorithms MLspike and CASCADE, and found each to have limitations in dealing with inhibited neurons. Among these spike inference algorithms, FOOPSI, from CaImAn, performed the best on inhibited neurons, but even this algorithm inferred spurious spikes upon the return of the fluorescence signal to baseline. As such, new approaches will be needed before spikes can be sensitively and accurately inferred from calcium data in inhibited neurons. We further suggest avoiding data analysis approaches that, by assuming non-negativity, ignore inhibited responses. Instead, we suggest a first exploratory step, using k-means or PCA for example, to detect whether meaningful negative deviations are present. Taking these steps will ensure that inhibition, as well as excitation, is detected in calcium imaging datasets.

## Introduction

The advent of Genetically Encoded Calcium Indicators (GECI) has transformed the field of neuroscience by allowing the imaging of activity across large populations of neurons (Nakai et al., 2001; Pologruto et al., 2004; Tian et al., 2009), and these methods are now being integrated in other fields of biology (Balaji et al., 2017; Shannon et al., 2017; Stevenson et al., 2020). A concurrent boom in microscopy techniques has allowed the rapid volumetric imaging of these populations, *in vivo*, in models including larval zebrafish (Wyart et al., 2009; Ahrens et al., 2012; Constantin et al., 2020; Vanwalleghem et al., 2020); flies (Wang et al., 2003; Suh et al., 2004), and rodents (Chen et al., 2012; Cai et al., 2016; Klioutchnikov et al., 2020). The vast datasets produced by this approach have driven the development of computational tools designed to extract and process activity information from populations of neurons (Mukamel et al., 2009; Freeman et al., 2014; Pachitariu et al., 2017; Giovannucci et al., 2019; Stringer and Pachitariu, 2019). A common assumption in most of these modern computational tools is the non-negativity of the GECI's signal.

However, negative deviations from the fluorescence baselines have been observed, and assumptions of non-negativity may cause the omission or misinterpretation of GECI data from populations with such negative deviations (Galizia et al., 2010; Munch and Galizia, 2017; Favre-Bulle et al., 2018; Marquez-Legorreta et al., 2019; Zimmerman et al., 2019). With the slow rise and decay of GECI probes, on the order of hundreds of milliseconds, a long-term average firing rate above 1 Hz would be convolved as a high fluorescence baseline. Such tonic activity can be found in vestibular neurons, even at rest (Shimazu and Precht, 1965; Cullen and McCrea, 1993), and in the primary visual cortex neurons (Baddeley et al., 1997) among a great many others. Notably, inhibition of tonically active neurons has been observed with electrophysiology in vestibular neurons (Shimazu and Precht, 1966), Purkinje cells (Tian et al., 2013), and distributed across the brain in response to stimulus-driven decisions (Steinmetz et al., 2019). Such inhibition of tonic neurons, convolved by the slow GECI kernels, translate to negative deviations from baseline as we and others have observed (Favre-Bulle et al., 2018; Zimmerman et al., 2019).

Many tools for GECI analysis include methods for inferring the spike train that generated the observed fluorescence signal, and again most of these spike deconvolution algorithms assume non-negativity (Vogelstein et al., 2010; Pachitariu et al., 2018). For example, the spikefinder online challenge had this implicit assumption in the datasets offered to the community (Theis et al., 2016), and their best performing algorithms were based on convolutional neural networks. This supervised approach, however, would miss inhibited response profiles as they have been trained on datasets with no negative deviation in the fluorescent traces.

Finally, this non-negative assumption is built into popular approaches for interpreting the patterns of activity across populations of neurons. For example, Non-negative Matrix Factorization (NMF), not to be confused with CNMF that is used to extract fluorescent traces from the videos (Pnevmatikakis et al., 2016), has been used as a dimensionality reduction or clustering tool on the fluorescent traces of individual neurons (Freeman et al., 2014; Mu et al., 2019; Torigoe et al., 2019). The NMF approach, when applied on extracted neuronal activity data normalized with z-scoring or ΔF/F_0_, discards negative deviations from the baseline fluorescent signal. Another approach that we and others have used, the binarization of the data based on a threshold of activity to generate “bar codes” of the brain activity, also has an intrinsic non-negative assumption (Kubo et al., 2014; Naumann et al., 2016; Heap et al., 2018; Daviu et al., 2020; Etter et al., 2020). Other threshold-based approaches, or even data cleaning steps, run the risk of discarding all negative deviations from baseline, biasing conclusions drawn from the dataset to exclude inhibition from the modeled system.

In summary, we find this non-negative assumption at all levels of calcium imaging analysis, from the extraction of fluorescence traces to spike inference and analyses of populations' dynamics. Our goal here was to assess how the most popular calcium imaging analyses responded to negative deviations from the baseline, including whether or not each approach was sensitive to traces that were typical of inhibitory signals in neural networks. We also hope to spark a discussion on how these assumptions may have biased past studies, and may continue to bias future work using GECIs.

## Materials and Methods

The imaging data came from Favre-Bulle et al. (2018). Briefly, experiments were carried on 6 day post-fertilization (dpf) *nacre* mutant zebrafish (Danio rerio) larvae of the Tüpfel long fin strain carrying the transgene *elavl3:H2B-GCaMP6s* (Chen et al., 2013). The larvae were immobilized in 2% low melting point agarose (Progen Biosciences, Australia) and imaged using a diffuse digitally scanned light-sheet microscope (Taylor et al., 2018) while an optical trap was applied to the otolith to simulate acceleration (Favre-Bulle et al., 2017, 2018, 2019, 2020). All procedures were performed with approval from the University of Queensland Animal Welfare Unit in accordance with approval SBMS/378/16/ARC.

Artificial datasets were generated using the Neural Anatomy and Optical Microscopy simulation toolbox (Charles et al., 2019). We used the parameters for nuclear simulation with GCaMP6f default (see Table 1). To simulate inhibited neuronal responses, we randomly attributed a spike number from a Poisson distribution (λ of 1, based on; Baddeley et al., 1997) to each 200 ms time window of 10 to 20 percent of all simulated neurons (since ~20% of neurons were inhibited when observed by Steinmetz et al., 2019). We then set a time frame of 0.2 to 5 s of inhibition (0 spikes), which was used to simulate the neuronal activity and generate movies that were processed with the tools below. For the mixed activity, we used a similar approach on the second half from the time series of 20% of the neurons in the simulated dataset.

**Table 1:** Parameters used for the simulation of calcium datasets.

**Frame rate**	**Simulated volume**	**Radius nuclei**	**τ of GECI**	**Time points**
5 Hz	90 × 90 × 50	5.9 m	1.5	1,000

For fluorescence extraction and spike inference, we benchmarked the most cited calcium imaging toolboxes: suite2p (suite2p, version 0.8.0, RRID:SCR_016434) (Pachitariu et al., 2017), CaImAn version 1.8 (Giovannucci et al., 2019), and the PCA/ICA approach CellSort (Mukamel et al., 2009). We did not simulate motion, and as such did not use the registration algorithms included in either suite2p or CaImAn. The parameters used for each of these approaches can be found in the github repository. Briefly, for suite2p we used the sourcery roi extraction, with a τ of 2, frame rate of 5, diameter of neurons (4,6), threshold scaling of 0.5 and a high pass of 50. For CaImAn, we used the CNMFe implementation which shows a better accuracy for background estimation than CNMF and should avoid the risks of spurious negative deviations due to background subtraction (Zhou et al., 2018). The parameters for CaImAn were a τ of 2, frame rate of 5, a gSig of 4 and autoregressive order of 2. For the deep-learning spike inference method CASCADE, we used the Universal_5Hz_smoothing200ms pretrained model to infer the spikes on our dataset (Rupprecht et al., 2020). For MLspike, we used τ = 2, dt = 0.2, pnonlin = [0.55 0.03] as suggested for GCaMP6f in Deneux et al. (2016).

For the analysis of the responses, we used MATLAB (R2018b, RRID: SCR_001622). ΔF/F_0_ was computed as in Akerboom et al. (2012). We used the non-negative matrix factorization function nnmf with 15 factors to reanalyze the data from Favre-Bulle et al. (2018). We used the correlation coefficients tools from MATLAB to compute the 2-dimensional correlation between the regions of interest (ROIs) and the ideal components, as well as between the traces or spikes and the ideal traces or spikes.

Statistical tests and plotting were done in Graphpad Prism (8.4.3, RRID:SCR_002798), using ordinary ANOVA with Tukey's multiple comparison test.

All the code used to generate and analyze the data can be found on github.com/Scott-Lab-QBI/NegativeCalciumResponses.

## Results

### Real Data

**Figure 1:**
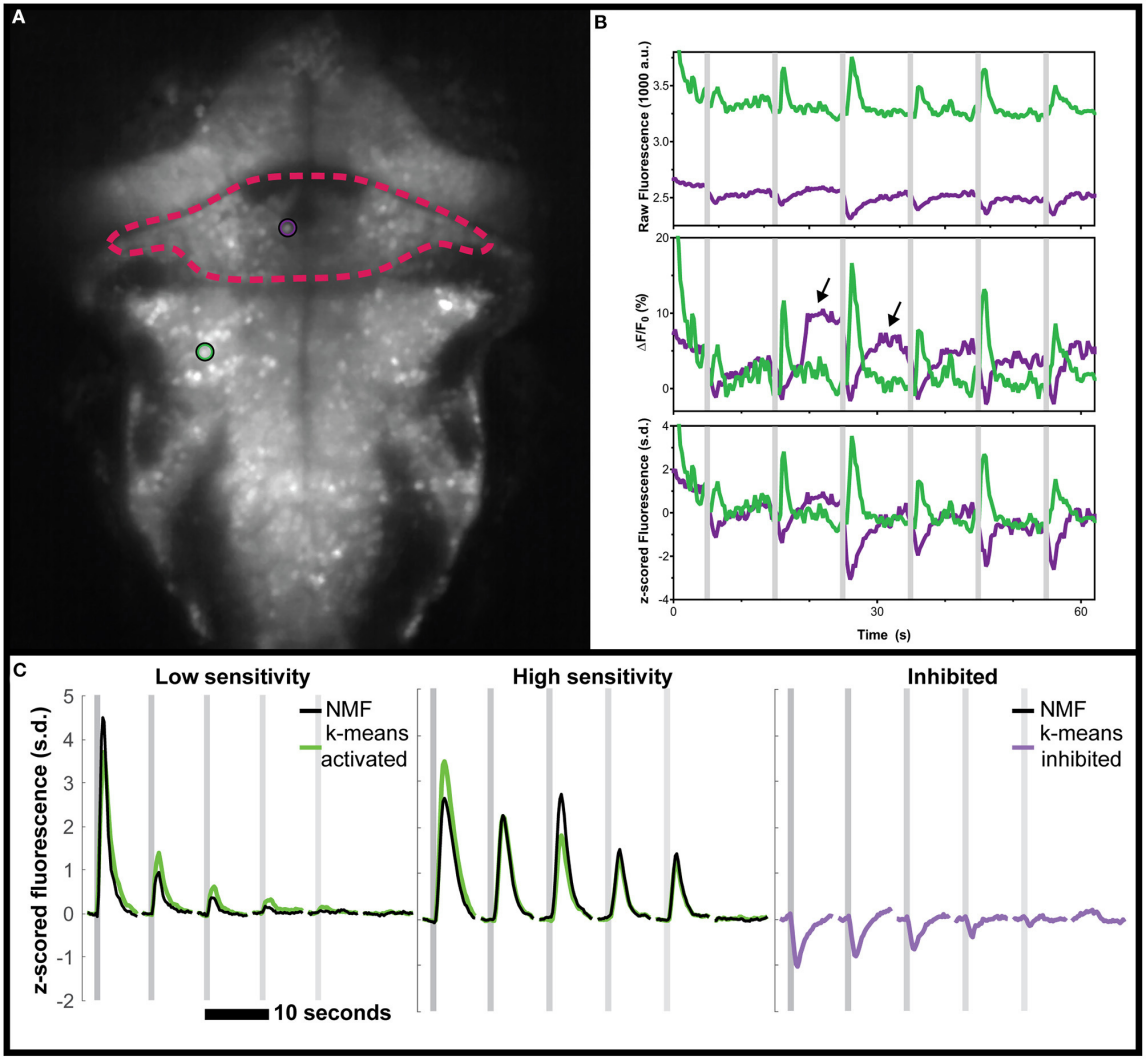
Negative deviations from baseline in real data from the cerebellum of zebrafish, and performance of various analysis tools. **(A)** Mean fluorescence image of a 6 dpf zebrafish expressing nuclear-targeted GCaMP6s (Chen et al., 2013). The cerebellum is outlined in red, and an inhibited neuron is indicated with a magenta circle. The green circle indicates an activated neuron in the hindbrain. **(B)** Time traces of the raw (top), ΔF/F_0_ (middle), or z-scored (bottom) fluorescence for these two neurons, in their respective colors. Arrows indicate positive deviation artifacts resulting from the cessation of inhibition on the inhibited neuron. **(C)** Comparisons between the clusters identified using k-means (green for activated, magenta for inhibited) and those identified with NMF (black). No inhibited cluster was identified by NMF. Gray shaded areas indicate the time of vestibular stimulation (Favre-Bulle et al., 2018), with a progression from strong to weak stimuli across the stimulus train.

First, we reanalyzed a zebrafish dataset from our previous study of vestibular processing in which we identified inhibited responses in hundreds of neurons across the thalamus and cerebellum (Favre-Bulle et al., 2018). For the analysis presented here, we focus on two representative neurons from the cerebellum and hindbrain of a larval zebrafish (Figure 1A) as larvae were subjected to vestibular stimuli (Figure 1B, shaded areas). As seen in the raw data (Figure 1B, arrows), we observe negative deviations from baseline during stimulation (Figure 1B, magenta traces), as well as positive responses (Figure 1B, green).

Our first observation was that the classical ΔF/F_0_ approach with a moving baseline window (Akerboom et al., 2012) creates positive artifacts following negative deviation from baseline as seen in Figure 1B (arrows). These positive artifacts could be construed as actual responses by some approaches, since they peak at the same level as the actual responses (magenta traces with arrows vs. adjacent green traces in Figure 1B). In the ΔF/F_0_ trace, the results do not correlate as well for the (magenta) inhibited neuron (ρ = 0.599) when compared to the (green) activated neuron (ρ = 0.979). However, the z-scored trace was perfectly correlated to the raw trace (ρ = 1) for both neurons. As such, we recommend the use of z-score as a normalization of calcium traces, and we will use this normalization in the following analysis.

Beyond these artifacts, there was the concern that popular data analysis methods could miss inhibited response profiles. NMF has been used to analyze larval zebrafish calcium imaging data (Mu et al., 2019; Torigoe et al., 2019), so we tested this method on the same vestibular dataset from our group (Favre-Bulle et al., 2018). As can be seen (Supplementary Figure 1), the NMF approach failed to identify responses resembling the inhibited cluster identified by k-means while the other (non-negative) clusters were found with a high correlation (ρ = 0.92, ρ = 0.94, respectively, Figure 1C).

The major limitation of this analysis was that it lacked a ground truth, making it impossible to judge whether outputs from apparently successful approaches actually reflected physiology. To solve this problem, we turned to simulated data for which we control the ground truth.

### Simulated Data

**Figure 2:**
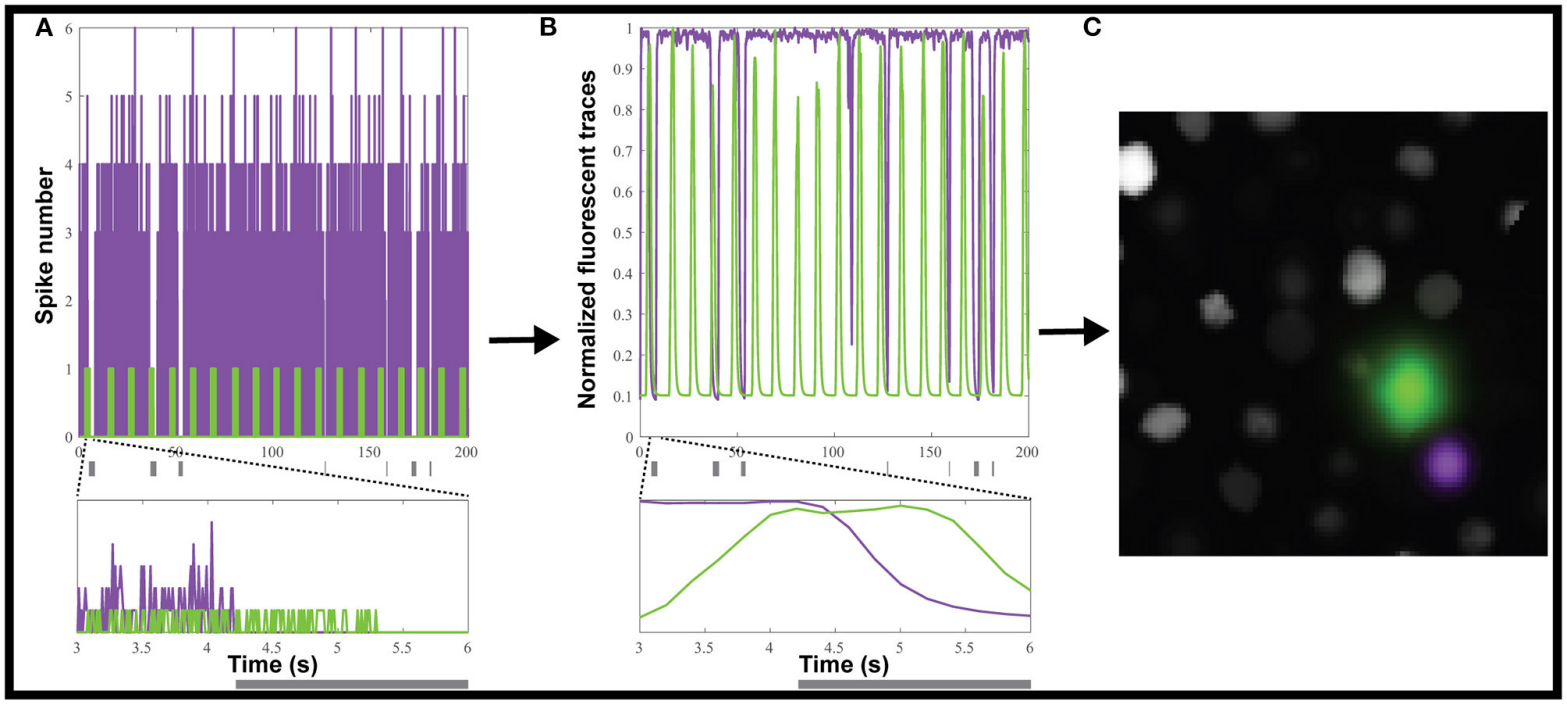
Creating simulated calcium imaging datasets. **(A)** An example dataset of simulated activity, showing spike numbers for one neuron (green) activated and one (magenta) inhibited by a hypothetical stimulus (gray rectangles). **(B)** The spike trains are convolved with a GCaMP6f kernel and noise to generate fluorescence traces. **(C)** The simulated neuronal activity was used to create an artificial movie as captured by a microscope.

We used the Neural Anatomy and Optical Microscopy (NAOMi) Simulation toolbox (Charles et al., 2019) to generate 10 datasets of simulated nuclear-targeted GCaMP6f data, as described in the Materials and Methods. Briefly, each dataset contained about 90 neurons, and for each, we randomly selected either 10 or 20% of the neurons to be inhibited. For each inhibited neuron, we simulated tonic firing, based on an observed Poisson distribution (Baddeley et al., 1997), which was randomly interrupted for 0.2 to 5 s to simulate inhibition (Figure 2A). We chose a random inhibition pattern as both suite2p and CaImAn depend on the correlation between pixels to generate the ROIs, and we wanted to make the inhibited neurons as easy to identify as possible, since most methods depend on local correlations to identify the neurons. The simulated spiking (Figure 2A) was then convolved with a GCaMP6f kernel to simulate neural activity (Figure 2B), which was then used to generate movies using NAOMi (Figure 2C). As most simulated neurons would be below the detection threshold, we used NAOMi to output the ideal responses corresponding to what would be detected with a microscope. While other algorithms occasionally identified additional neurons, the effect was marginal (<1%), so we decided to use the ideal responses as ground truth for the sake of simplicity (Charles et al., 2019).

**Figure 3:**
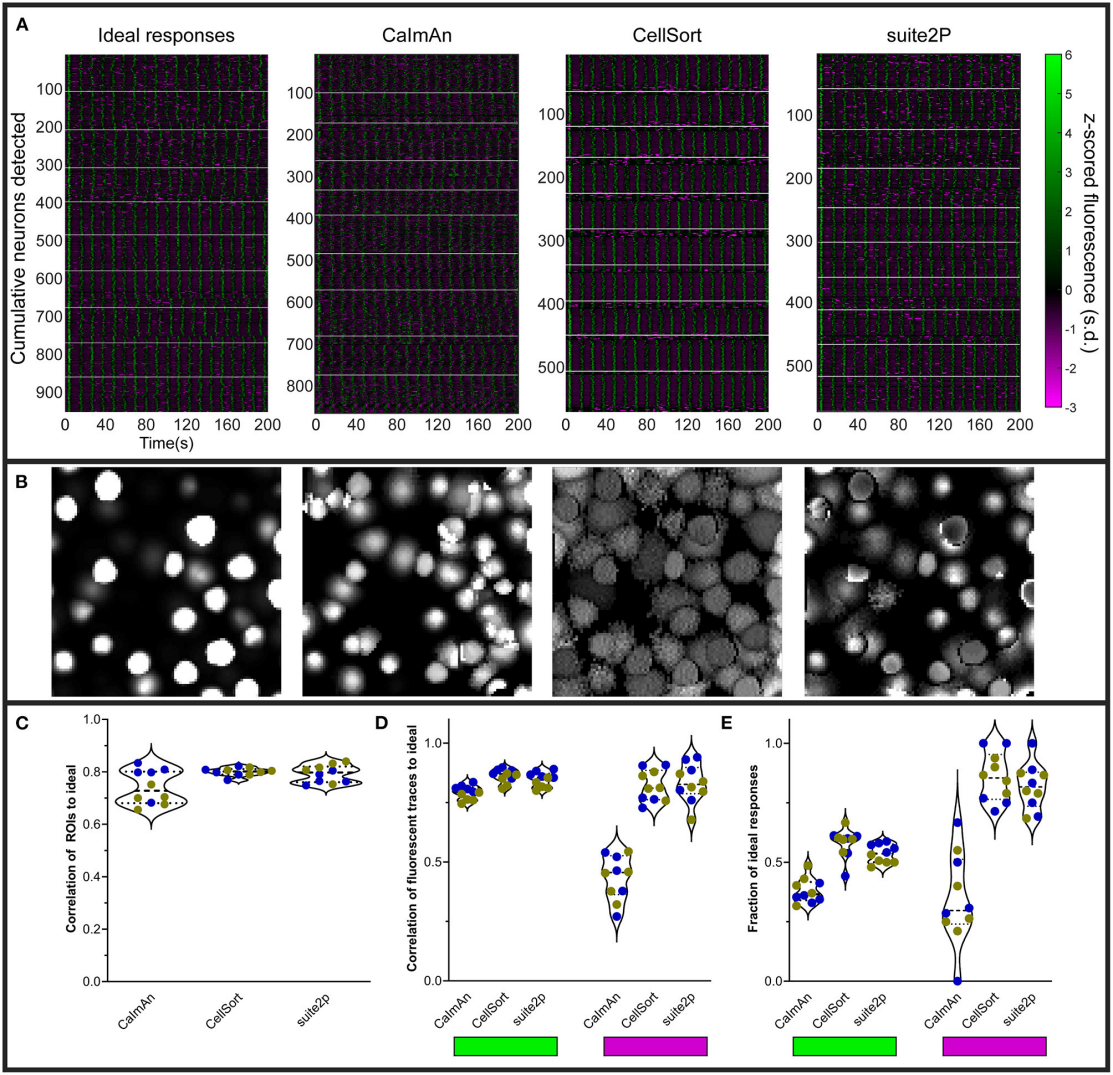
Various analyses' performances on simulated data. **(A)** Raster plots of ideal responses from NAOMi, and extracted fluorescence traces from CaImAn, CellSort, and suite2p. All the fluorescent traces were z-scored from−3 to 6 s.d. White horizontal lines separate the individual datasets. **(B)** Segmentation of the regions of interest (ROIs) by each algorithm, as for the raster plots in **(A)**, for one representative dataset. **(C)** Quantification of the correlation between the ROIs identified by each of the three algorithms and the ideal ROIs. Symbol color indicate the percentage of inhibited neurons (*n* = 5 datasets with 10% inhibited neurons in blue, and *n* = 5 datasets with 20% inhibited neurons in yellow). **(D)** Average maximum correlations between the traces identified by each algorithm and the ideal responses for the activated neurons (left, green rectangle) and the inhibited neurons (right, magenta rectangle). **(E)** Fraction of the ideal responses identified with a correlation above 0.5 by the three algorithms for the activated neurons (left) and the inhibited neurons (right).

Each fluorescence dataset was processed through suite2p (Pachitariu et al., 2017), CaImAn (Giovannucci et al., 2019), or CellSort (Mukamel et al., 2009), and the outputs for each approach were then analyzed in the same manner. We did not investigate whether the suite2p default classifier or the CaImAn components evaluation would exclude inhibited neurons, and as such, we kept all the ROIs either algorithm identified. The raster plots of the ten datasets (Figure 3A) show that CaImAn identifies the highest number of ROIs, with CellSort and suite2p identifying a similar number of ROIs (Ideal = 94.3 ± 4.7, CaImAn = 84.7 ± 14.4, CellSort = 55.5 ± 3.8, suite2p = 56.1 ± 4.7).

The segmentation of the simulated fluorescent movies gave good results for all three algorithms, with well-defined regions of interest that correlated well with the ideal ROIs (Figures 3B,C, ρ_CaImAn_ = 0.74 ± 0.06, ρ_CellSort_ = 0.80 ± 0.02, ρ_suite2p_ = 0.79 ± 0.03). We then correlated the ideal traces of activated or inhibited simulated neurons to the traces extracted by each algorithm, and for each dataset, we averaged the maximum correlations to each ideal trace (Figure 3D). All three algorithms succeeded in extracting the relevant traces for the activated neurons (Figure 3D, left, indicated by green bar), but CellSort and suite2p outperformed CaImAn for the inhibited traces (ρ_CaImAn_ = 0.43 ± 0.09, ρ_CellSort_ = 0.82 ± 0.07, ρ_suite2p_ = 0.83 ± 0.08, Figure 3D, right, magenta).

To assess the proportion of true positives, we identified the ideal fluorescent trace to which each ROI's fluorescent trace best correlated. We only counted the unique ROIs that passed a 0.5 correlation cut-off, as all algorithms over-segment some of the sources in duplicated fluorescent traces (Charles et al., 2019). When comparing the proportions of identified ideal activated neurons, CellSort outperformed suite2p slightly, followed by CaImAn (proportions of 0.38 ± 0.05_CaImAn_, 0.58 ± 0.06_CellSort_, and 0.54 ± 0.04_suite2p_, Figure 3E left). For inhibited neurons, CellSort outperformed suite2p slightly again, but the divide with CaImAn grew (proportions of 0.34 ± 0.19 _CaImAn_, 0.86 ± 0.10 _CellSort_ and 0.82 ± 0.10 _suite2p_, Figure 3E, right). All algorithms seemed insensitive to the ratio of inhibited neurons presented, as we saw no difference in those metrics between datasets with 10 vs. 20% inhibited neurons.

These results are lower than the results from Charles et al. (2019), who found that both CaImAn and suite2p outperformed CellSort (proportions of 0.71, 0.69 and 0.33, respectively). One possible explanation for the difference is that our use of nuclear-targeted GCaMP simulations, like our real datasets, may favor CellSort.

### Spike Inference From Simulated Calcium Traces

**Figure 4:**
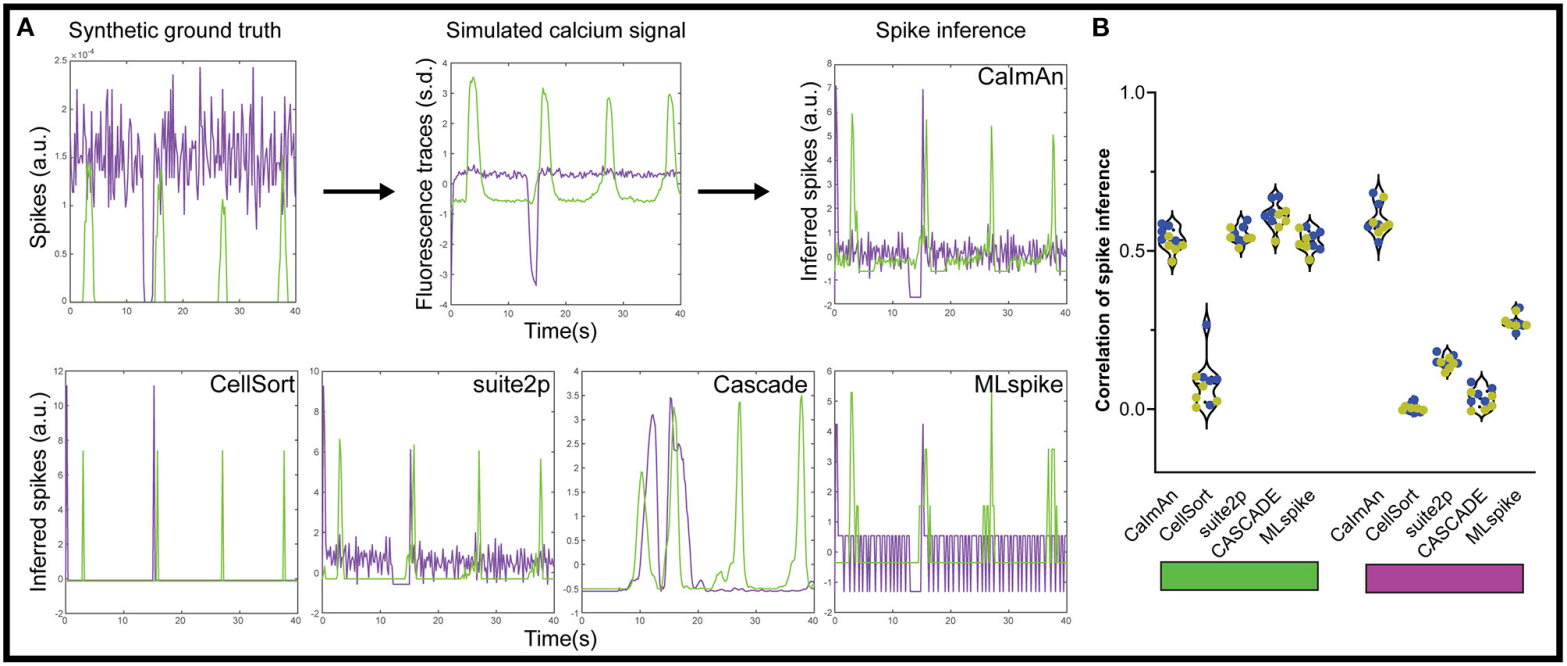
Spike inference from simulated calcium traces. **(A)** We applied each of the five spike inference algorithm on simulated GECI fluorescence data generated from a synthetic ground truth. The inferred spikes were then compared to the synthetic ground truth using a correlation. We show example inferred spikes from each algorithm, as well as the synthetic ground truth and simulated fluorescence. **(B)** Correlation between the inferred spikes from the simulated calcium traces and the actual spikes for the activated neurons (left, green rectangle) and the inhibited neurons (right, magenta rectangle). Each datapoint represents the performance on one simulated dataset (*n* = 5 datasets with 10% inhibited neurons in blue, and *n* = 5 datasets with 20% inhibited neurons in yellow).

In theory, inferring the spike trains responsible for calcium traces is one way to improve the temporal resolution, as you get rid of the convolved GCaMP kernel, but the frame rate of acquisition often makes such deconvolution impractical and unreliable. Each of the above algorithms offers some form of spike inference (Figure 4A), and multiple other approaches have been proposed during an online challenge (Berens et al., 2018). CaImAn offers multiple options for spike inference, among which we selected their fast non-negative deconvolution (FOOPSI) method (Vogelstein et al., 2010). For suite2p, we used the Online Active Set method to Infer Spikes (OASIS) (Friedrich et al., 2017). We also tested a recent spike inference method based on deep learning, CASCADE, which offers universal pre-trained models (Rupprecht et al., 2020) and a maximum likelihood approach to the probable spiked train, MLspike (Deneux et al., 2016).

Using this approach, we tested how accurate each spike detection algorithm was on our datasets. To avoid any confounding issues from the detection algorithm, we used the ideal calcium responses as the basis for the spike detection. Based on our results with the moving baseline of ΔF/F_0_ (Figure 1B), we also did not pre-process the data for the spike inference with suite2p, and used a global minimum to normalize for CASCADE and MLspike. The CellSort deconvolution approach had limited success with both activated and inhibited neurons (ρ_CellSort_ = 0.08 ± 0.08, and ρ_CellSort_ = 0.003 ± 0.013 respectively, Figure 4B, Supplementary Figure 2). The more recent CaImAn and suite2p did well for the activated neurons, (ρ_CaImAn_ = 0.53 ± 0.04, ρ_suite2p_ = 0.55 ± 0.03), but CaImAn outperformed suite2p for inhibited neurons (ρ_CaImAn_ = 0.60 ± 0.05, ρ_suite2p_ = 0.37 ± 0.03). The universal model of CASCADE performed better than the rest on the activated neurons (ρ_CASCADE_ = 0.66 ± 0.03), but worse on the inhibited neurons (ρ_CASCADE_ = 0.03 ± 0.03). MLspike performed slightly worse than the above algorithms on the activated neurons (ρ_MLspike_ = 0.53 ± 0.03), but was intermediate on the inhibited neurons (ρ_MLspike_ = 0.28 ± 0.02).

Those performances were also tested in a scenario containing neurons with a mixture of activation and inhibition (Supplementary Figure 3). In that scenario there was little difference between the activated neurons and the mixed activity neurons with the different algorithms for ROI detections. The spike inference results were slightly worse across the board, but not as strongly as in Figure 4.

## Discussion

In this study, we show that the often implicit assumption of non-negativity for calcium imaging data can lead to missing real responses from inhibited neurons. Current approaches run the risk of missing a significant fraction of responses at every step of the analysis pipeline, including cleaning the data, processing, feature extraction, dimensionality reduction, and clustering.

We have shown these negative deviations exist in real data from zebrafish, as we previously observed (Figure 1; Favre-Bulle et al., 2018), and as observed in mice (Steinmetz et al., 2019) and flies (Galizia et al., 2010; Munch and Galizia, 2017).

Hyperpolarization is well-known to decrease GCaMP signals in cells that are partially active at resting potential and that can be further inactivated by hyperpolarization (Zhao et al., 2018). We speculate that the negative deflections that we observed in GCaMP6 signals of our real dataset (Figure 1) are, based on the spatial location and activity of the ROIs, Purkinje cells. In zebrafish, Purkinje cells receive excitatory inputs from granule cells and climbing fibers, and inhibitory inputs from stellate interneurons. Their cell bodies are located within one of the most superficial layers of the cerebellum, and hence would be the first cells to be optically sectioned from the dorsal orientation. Purkinje cells display bistable spontaneous activity, where they switch between the steady production of tonic or depolarising “up” spikes and short bursts of intermittent activity or hyperpolarizing “down” states (Sengupta and Thirumalai, 2015). Therefore, the negative defections in GCaMP6 signals that we observed in our real dataset in Figure 1 could be tonically-active Purkinje cells in the superficial layers of cerebellum that have toggled to their climbing-fiber induced bursting or “down” state (Engbers et al., 2013), which would produce negative voltages.

We have demonstrated that a moving baseline, such as for ΔF/F_0_, may create artifacts in inhibited neurons, which may lead to the generation of spurious positive signals. Finally, inhibited responses, when normalized, can also be lost when using NMF or thresholding approaches to analyze and visualize the data (Figure 1C). Even pixel-wise NMF approaches such as Thunder could miss inhibited responses, if they include a preprocessing step such as ΔF/F_0_ (Freeman et al., 2014). It would be interesting to revisit the data from studies that used these approaches (Mu et al., 2019; Torigoe et al., 2019) to see whether inhibited neurons are present in the datasets. We suggest that an initial unbiased step of data exploration of the dataset should be performed to ensure that no inhibited responses are present before pursuing steps including the above methods that assume non-negativity. Principal component analysis, or other dimensionality reduction tools, could be used to explore the data in the case of spontaneous activity or complex stimuli. Alternatively, for stimulus-driven activity, a correlation or linear regression should reveal any neuronal activity that deviates negatively from baseline.

By using simulated data (Figure 2), we tested how reliably CellSort, suite2p, and CaImAn could detect inhibited neurons in a calcium imaging dataset. CellSort was the best algorithm in our specific analysis of nuclear-targeted GCaMP (Figure 3), which is at odds with other comparisons (Charles et al., 2019). However, both CaImAn and suite2p are better suited to larger datasets of thousands of neurons. Between these two approaches, suite2p outperformed CaImAn for the detection of activated responses both in terms of the fidelity of the extracted response (Figure 3D, mean difference of 0.056 and *p* = 0.0006) and the fraction of responses identified (Figure 3E, mean difference of 0.16 and *p* < 0.0001). For inhibited responses, suite2p largely outperformed CaImAn with more than twice the fraction of ideal inhibited responses recovered (mean difference = 0.47 and *p* < 0.0001). CellSort is a good option for smaller datasets as it requires an *a priori* estimate of the number of components (neurons) and does not perform as well at low SNR typical of endoscopic recordings (Resendez et al., 2016; Zhou et al., 2018). Among the currently available approaches, we therefore favor suite2p, or CellSort for smaller datasets, in order to recover the most inhibited responses from calcium imaging of neuronal activity.

As for the spike inference, the algorithm included with CellSort did poorly on both activated and inhibited neurons. MLspike was outperformed by suite2p and CaImAn performed similarly to one another with activated neurons, in line with published results (Pachitariu et al., 2018). However, for inhibited responses, suite2p's performance collapsed when using OASIS. CASCADE performed well on the activated neurons, but the lack of inhibited neurons in the training datasets mean it performed poorly when detecting our inhibited responses, as such the use of a more varied training dataset could improve its performance. Overall CaImAn, using FOOPSI, presents the best approach to infer spikes from inhibited neurons (Vogelstein et al., 2010). Several other methods of spike inference have been benchmarked (Berens et al., 2018), and it would be interesting to benchmark these with simulated inhibited neurons. Finally, we want to point out that all the spike inference algorithms mistakenly inferred a strong spiking probably/rate when the inhibition ended (Figure 4A, bottom), this would need to be accounted for in any downstream analysis of these inferences.

We saw no significant differences between simulated datasets with 10 or 20% inhibited neurons in any of the above metrics, showing that the proportion of inhibited neurons should not affect the detection of the activated neurons.

Overall, we suggest that the PCA/ICA approach, such as implemented in CellSort should be favored when dealing with smaller datasets, where the number of ROIs can be estimated before processing, and nuclear-targeted GECIs. For larger datasets however, we suggest using suite2p, which has worked well both with nuclear-targeted simulations in this study, and with a cytoplasmic GECI simulation (Charles et al., 2019). With regard to spike inference, the FOOPSI approach gave the best results, so we would favor this method when inferring spikes. In terms of data analysis, NMF or thresholding based on activity should be avoided before an unbiased analysis such as PCA, or k-means can be used to ensure the absence of relevant inhibited neurons.

Another way to address the issue of negative deviations is to change the calcium indicator used. For example, an inverse-response GECI (Zhao et al., 2018) has been specifically designed to easily visualize neuronal inhibition in flies, but then the activated neurons would be the ones deviating negatively from the baseline. A powerful alternative is the use of Genetically Encoded fluorescent Voltage Indicators (GEVIs), which directly report membrane potential (Akemann et al., 2010; Gong et al., 2015; Bando et al., 2019). Those GEVIs can be used to visualize action potentials with millisecond time resolution, their signal to noise ratio are constantly improving and they would offer an accurate measure of the actual spikes of the imaged neurons. However, the requirement of ~kHz imaging speed precludes their use for volumetric or whole-brain imaging with the current technologies.

Finally, GECIs allow the genetic targeting of the calcium indicator to subtypes of neuronal cells, providing information on the expected firing rate and behavior of the neurons (Scott et al., 2007; Forster et al., 2017). Alternatively, imaged neurons can be identified *post-hoc* using fixation and labeling (Lovett-Barron et al., 2017), which can be used to choose an analysis method that would be more appropriate if one expects negative deviations.

In summary, we have shown that assumptions of non-negativity can lead to the omission of real and simulated inhibited responses, and can produce spurious positive signals during the analysis of neural calcium imaging datasets. We have tested three popular and readily available approaches for analyzing such data, and provide recommendations for the best approaches to use when analyzing calcium imaging data that may contain inhibited signals.

## Data Availability Statement

The datasets generated for this study can be found in online repositories. The names of the repository/repositories and accession number(s) can be found at: doi: 10.14264/63584b3.

## Ethics Statement

The animal study was reviewed and approved by SBMS/378/16/ARC.

## Author Contributions

GV, LC, and ES contributed conception and design of the study and wrote sections of the manuscript. GV performed the statistical analysis. GV and LC wrote the first draft of the manuscript. All authors contributed to manuscript revision, read, and approved the submitted version.

## Conflict of Interest

The authors declare that the research was conducted in the absence of any commercial or financial relationships that could be construed as a potential conflict of interest.
