# Supplementary material for: Calcium Imaging and the Curse of Negativity
Source: Front Neural Circuits. 2021 Jan 6;14:607391. doi: 10.3389/fncir.2020.607391 (PMC7815594; doi:10.3389/fncir.2020.607391)
Supplement: Supplementary file 1 [file Data_Sheet_1.docx]

Supplementary Material

## Supplementary Figures


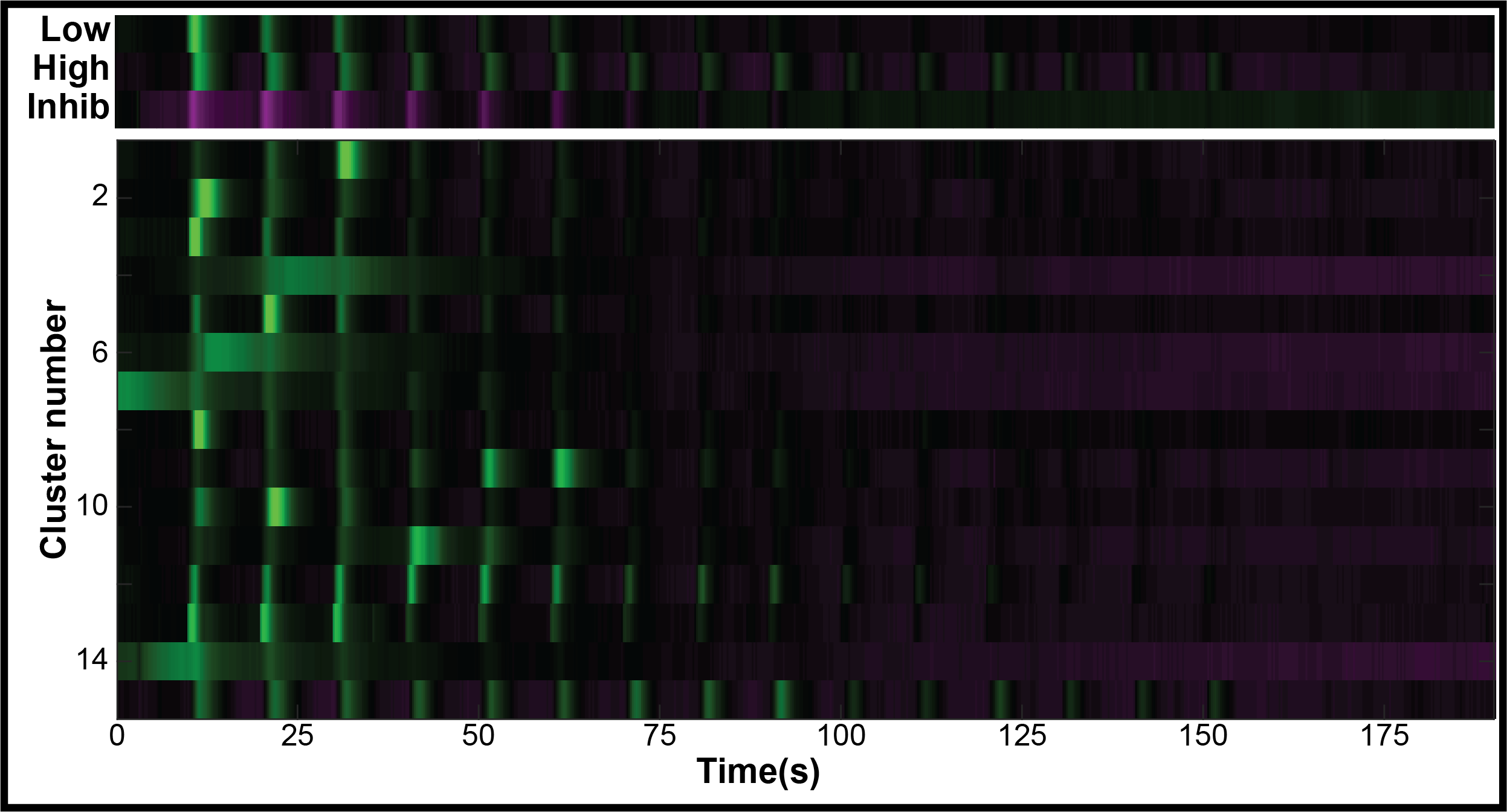
**Supplementary Figure 1: Clustering using Non-negative Matrix Factorization**

The data from (Favre-Bulle, Vanwalleghem et al. 2018) were clustered with an NMF approach, using the same number of clusters as the K-means used in the original paper. Of the 15 clusters, NMF identified the low sensitivity cluster (Cluster #3) and high sensitivity cluster (#15), but no inhibited cluster.

**
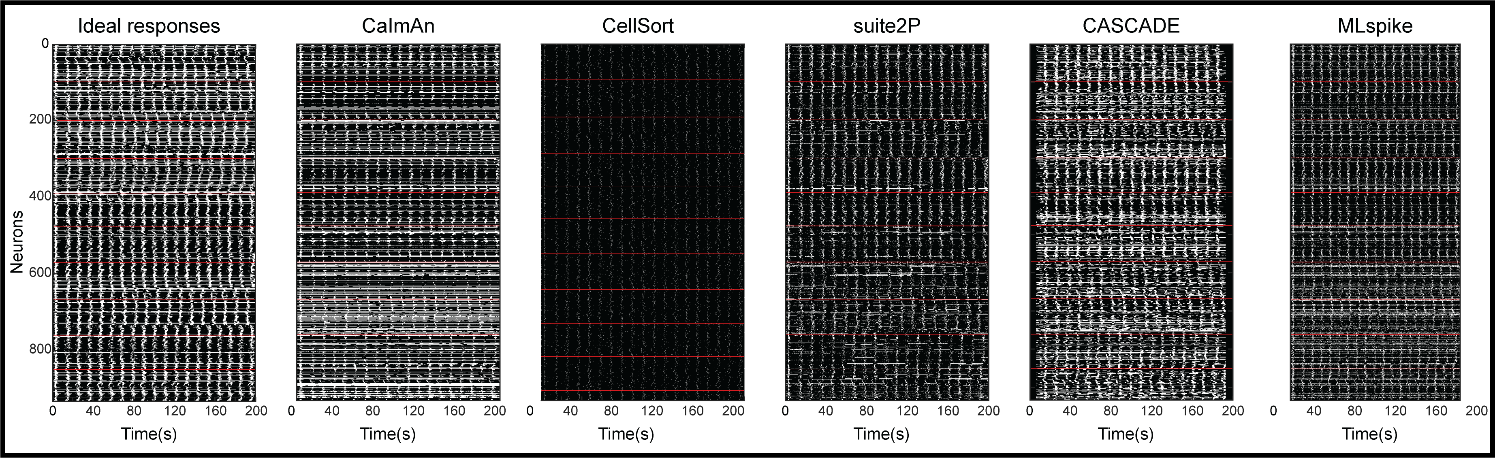
**

**Supplementary Figure 2: Inferred spike trains**

Binarized spike trains inferred from the ideal responses using the algorithms indicated above each panel.

**
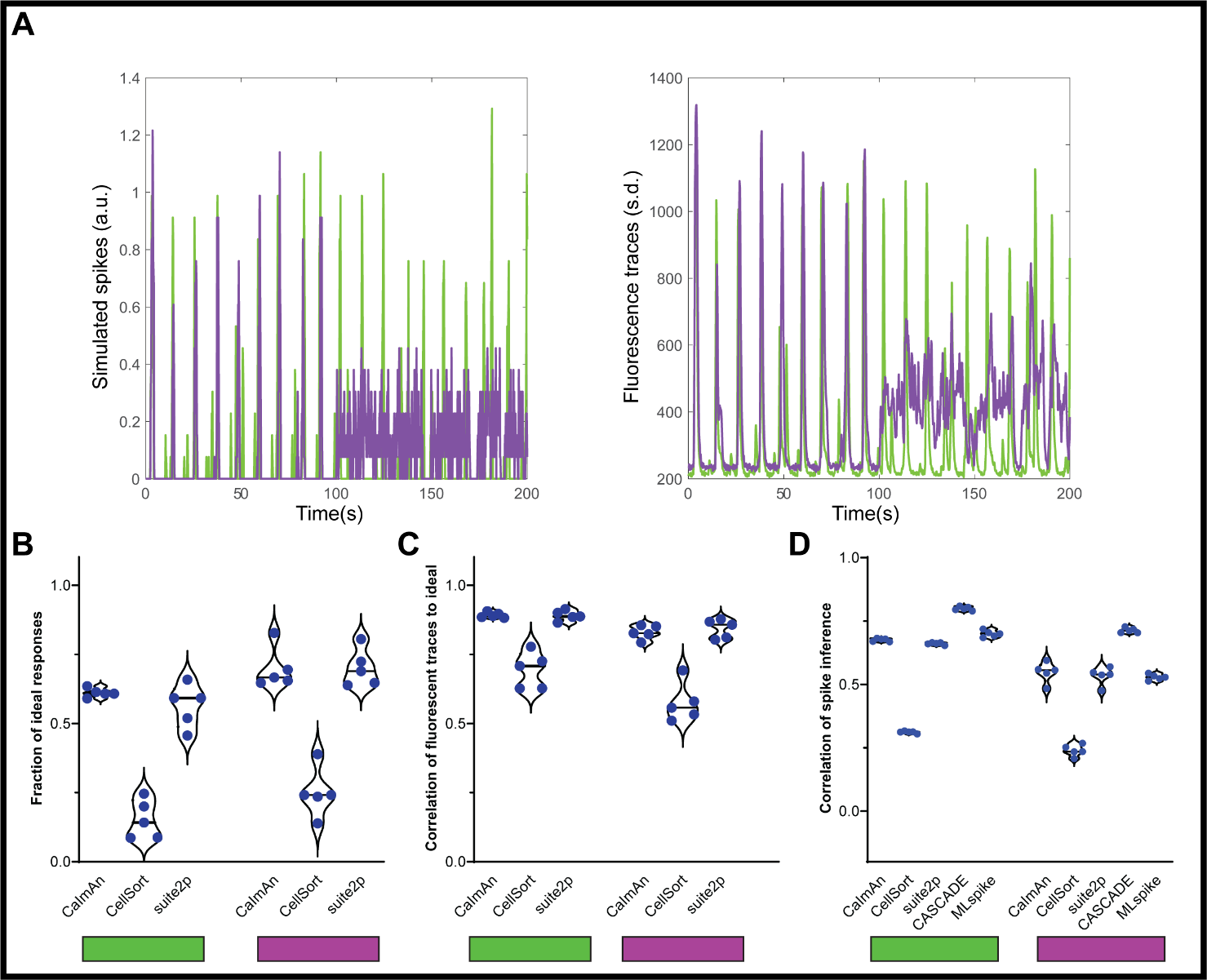
**

**Supplementary Figure 3: Effect on the algorithms from a mix of activated and inhibited activity**

(A) Representative spikes (left) and simulated fluorescence (right) from two neurons, in green is an activated neuron and in magenta is a representative inhibited neuron. (B) Fraction of the ideal responses identified (correlation above 0.5) by the three algorithms for the activated neurons (left, green rectangle) and the neurons with a mixture of activated and inhibited activity (right, magenta rectangle). (C) Average maximum correlations between the traces identified by each algorithm and the ideal responses for the activated neurons (left, green rectangle) and the mixed neurons (right, magenta rectangle). (D) Correlation between the inferred spikes from the simulated calcium traces and the actual spikes for the activated neurons (left, green rectangle) and the mixed neurons (right, magenta rectangle). Each datapoint represents the performance on one simulated dataset (n=5).
